# Stacking-based and improved convolutional neural network: a new approach in rice leaf disease identification

**DOI:** 10.3389/fpls.2023.1165940

**Published:** 2023-06-06

**Authors:** Le Yang, Xiaoyun Yu, Shaoping Zhang, Huanhuan Zhang, Shuang Xu, Huibin Long, Yingwen Zhu

**Affiliations:** School of Computer and Information Engineering, Jiangxi Agricultural University, Nanchang, China

**Keywords:** ensemble learning, stacking, convolutional neural network, machine learning, rice diseases

## Abstract

Rice leaf diseases are important causes of poor rice yields, and accurately identifying diseases and taking corresponding measures are important ways to improve yields. However, rice leaf diseases are diverse and varied; to address the low efficiency and high cost of manual identification, this study proposes a stacking-based integrated learning model for the efficient and accurate identification of rice leaf diseases. The stacking-based integrated learning model with four convolutional neural networks (namely, an improved AlexNet, an improved GoogLeNet, ResNet50 and MobileNetV3) as the base learners and a support vector machine (SVM) as the sublearner was constructed, and the recognition rate achieved on a rice dataset reached 99.69%. Different improvement methods have different effects on the learning and training processes for different classification tasks. To investigate the effects of different improvement methods on the accuracy of rice leaf disease diagnosis, experiments such as comparison experiments between single models and different stacking-based ensemble model combinations and comparison experiments with different datasets were executed. The model proposed in this study was shown to be more effective than single models and achieved good results on a plant dataset, providing a better method for plant disease identification.

## 1 Introduction

Crop diseases are primary agricultural issues worldwide because they occur often and can lead to significant crop yield reductions. Recently, automatic crop disease image recognition has received much attention because of food security concerns. This is a challenging research issue due to the complex natures of crop disease images, such as their cluttered field backgrounds and irregular illumination intensities (Chen et al., 2021). The appearance of various crop diseases adversely influences plant growth; when these crop diseases are not discovered early, they may have disastrous food security consequences (Picon et al., 2019). Especially in the case of rice, which is one of the world’s important grains, it is important to prevent diseases as an important means of improving rice production. However, there are many types of rice diseases, and disease symptoms possess complex and variable information. These diseases can only be accurately identified and diagnosed by visual inspections performed by professionally trained plant specialists, and although this method can suppress some disease epidemics, it requires a large workload, is costly and is not easy to promote. Therefore, a fast, inexpensive and effective method for crop disease identification is needed due to its important practical significance.

With the rapid development of computers and digital technology, a new generation of crop disease identification is on the horizon. Computer vision technology offers an interesting and attractive alternative for the serial monitoring of crop diseases because of its low price and visual and noncontact nature (Wells, 2016). Many works have used machine learning for the purpose of recognizing and classifying crop diseases (Pawiak et al., 2019; Hammad et al., 2020; Tuncer et al., 2020). For example, Singh et al. (2015) proposed a classifier using a support vector machine (SVM) for identifying rice leaf diseases by using a k-means clustering algorithm to segment leaf infection sites as classifier inputs; this approach eventually achieved a recognition rate of 82%. Gharge and Singh (2016) proposed a backpropagation neural network to classify soybean frog eyes, downy mildew and bacterial pustules with 93.3% accuracy using image enhancement techniques to separate infected clusters from leaves *via* a k-means segmentation algorithm. Kaur et al. (2018) used k-means to distinguish diseased leaves from healthy leaves and trained models using SVM classifiers to implement a semiautomatic system for identifying three soybean diseases, where a maximum average accuracy of 90% was achieved. Zhong et al. (2019) proposed a method based on FPCA and an SVM for solving the difficult problem potato disease localization and identification, and experiments showed that their method achieved great success with a recognition rate of 98%. Garcia et al. (2020) used an SVM classifier and the CIELab color space to identify the ripeness of tomatoes *via* a machine learning method using a 5-fold cross-validation strategy and achieved a recognition rate of 83.39% across 900 images.

Although the above methods have achieved good results, it is difficult to extract many features from images solely using machine learning methods, resulting in low accuracy, until a new technique, deep learning, was proposed; deep learning overcomes most digital image processing challenges and promises to identify a wide range of crop diseases. Currently, deep learning is also becoming increasingly used in the field of agriculture (Jiang et al., 2019; Jahanbakhshi et al., 2020; Pardede et al., 2020; Sugathan et al., 2020; Xie et al., 2020). Various studies have shown that deep learning networks can achieve good plant disease identification and classification results. Kawasaki et al. (2015) proposed a novel plant disease detection system with convolutional neural networks that achieved 94.9% accuracy in terms of cucumber disease identification using 4-fold cross-validation. Brahimi et al. (2017) used convolutional neural networks as learning algorithms to train on a dataset containing nine tomato diseases and used visualization methods to locate the disease sites with good results, yielding an accuracy of 99.18%. Cai et al. (2019) used convolutional neural networks to extract features such as the sizes, colors, textures and roundness of apples and then implemented an SVM to classify the ranks of Yantai apples, verifying the effectiveness of the convolutional neural network-SVM combination. Jiang et al. (2020) combined convolutional neural networks with SVM to identify rice leaf diseases using 10-fold cross-validation, achieving an average accuracy of 96.8%. Li et al. (2022a) ResNet and Inception V1 extracted the global features of the image, and then added WDBlock to the DCGAN generator. At the same time, SeLU activation function was used to improve the training stability of the network. Experiments have shown that FWDGAN can generate higher quality data and reduce the number of model parameters without compromising network performance. In the same year, Li et al. (2022b) proposed a tree image recognition system based on the Caffe platform and dual task Gabor. The Gabor kernel was introduced into CNN to extract frequency domain features of images with different scales and directions, thereby enhancing the texture features of leaf images and improving recognition performance. The training accuracy in complex backgrounds is 96%, achieving the goal of efficient and accurate recognition of quantities. Shundong Fang et al. (2022) used MFF blocks as the main structure, adopted a multi-channel feature fusion module of LC Block and RCblock, and added a hard coordination attention mechanism module to improve the recognition accuracy of the network. The final proposed HCA-MFFNet network for corn diseases has a recognition rate of 97.75%.

The network performance proposed in the above references is good, but there are similarities between some rice leaf diseases, making it difficult for neural networks to recognize them. Therefore, this study using the stacking integrated learning method, the proposed base learner integrates four convolutional neural networks, an improved AlexNet, an improved GoogLeNet, ResNet50 and MobileNetV3, for extracting rice leaf disease features, and an SVM is selected as the sublearner for disease classification and prediction; the accuracy of the proposed approach reaches 99.69%. Compared with the existing method, this method has the following four main contributions:

1. In this study, a stacking-based ensemble learning model is proposed for efficient and accurate identification of rice leaf diseases.
2. Combine convolutional neural network with support vector machine (SVM) to improve the disease identification effect of rice leaves.
3. Help improve model performance by increasing attention mechanisms and inserting residual networks into the model as convolutional layers.
4. The Leaky function is used instead of the RELU function to improve the extraction of disease characteristics of rice leaves by the model and reduce the sparsity of the RELU function.

The rest of this article is structured as follows. In Section 2, image acquisition and image preprocessing are mainly introduced. Section 3 introduces the construction process of the proposed model in detail, mainly discussing the method of the proposed model. These experiments are described in detail in Section 4, providing a large number of experiments to probe the performance of the proposed method and analyze the experimental results. Finally, section 5 summarizes the document and presents future work.

## 2 Materials and methods

### 2.1 Image acquisition

**Figure 1:**
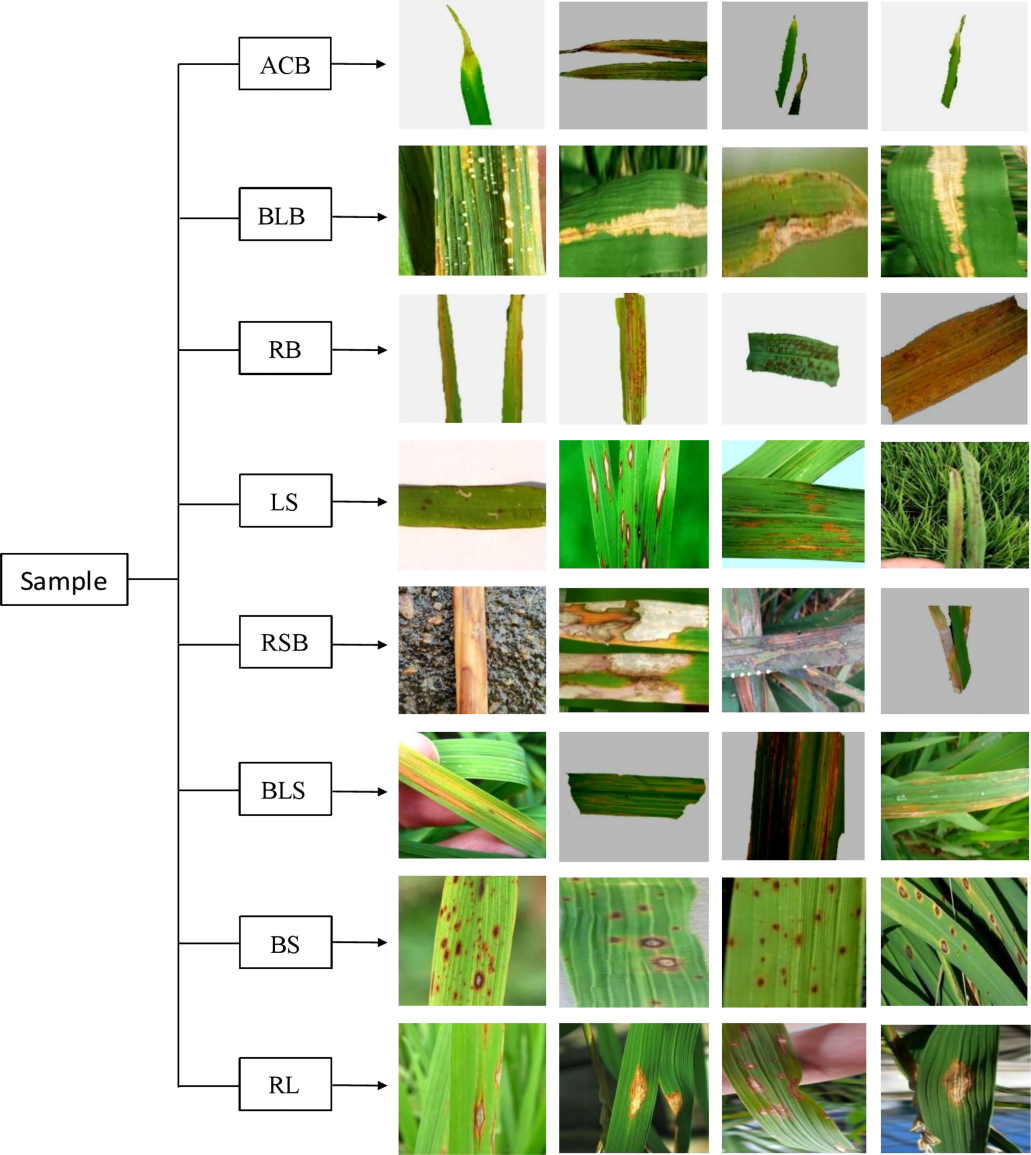
Partial leaf disease map of rice.

In this study, images of diseased rice leaves were taken with a Canon EOS 6D MarkII digital camera at the Agronomy Experiment Station of Jiangxi Agricultural University, and 36 images were identified and screened by experts. An additional 1086 images were obtained through search engines as well as from the public dataset Kaggle, for a total of 1122 rice leaf disease images. These images contained eight kinds of diseases, Aphelenchoides besseyi, bacterial leaf blight, red blight, leaf smut, rice sheath blight, bacterial leaf streaks, brown spots and rice blasts, and some of these rice leaf disease images are shown in **Figure 1**. Among them, ACB is Aphelenchoides besseyi, BLB is bacterial leaf blight, RB is red blight, LS is leaf smut, RSB is rice sheath blight, BLS is a bacterial leaf streak, BS is a brown spot, and RL is a rice blast.

### 2.2 Image processing

**Figure 2:**
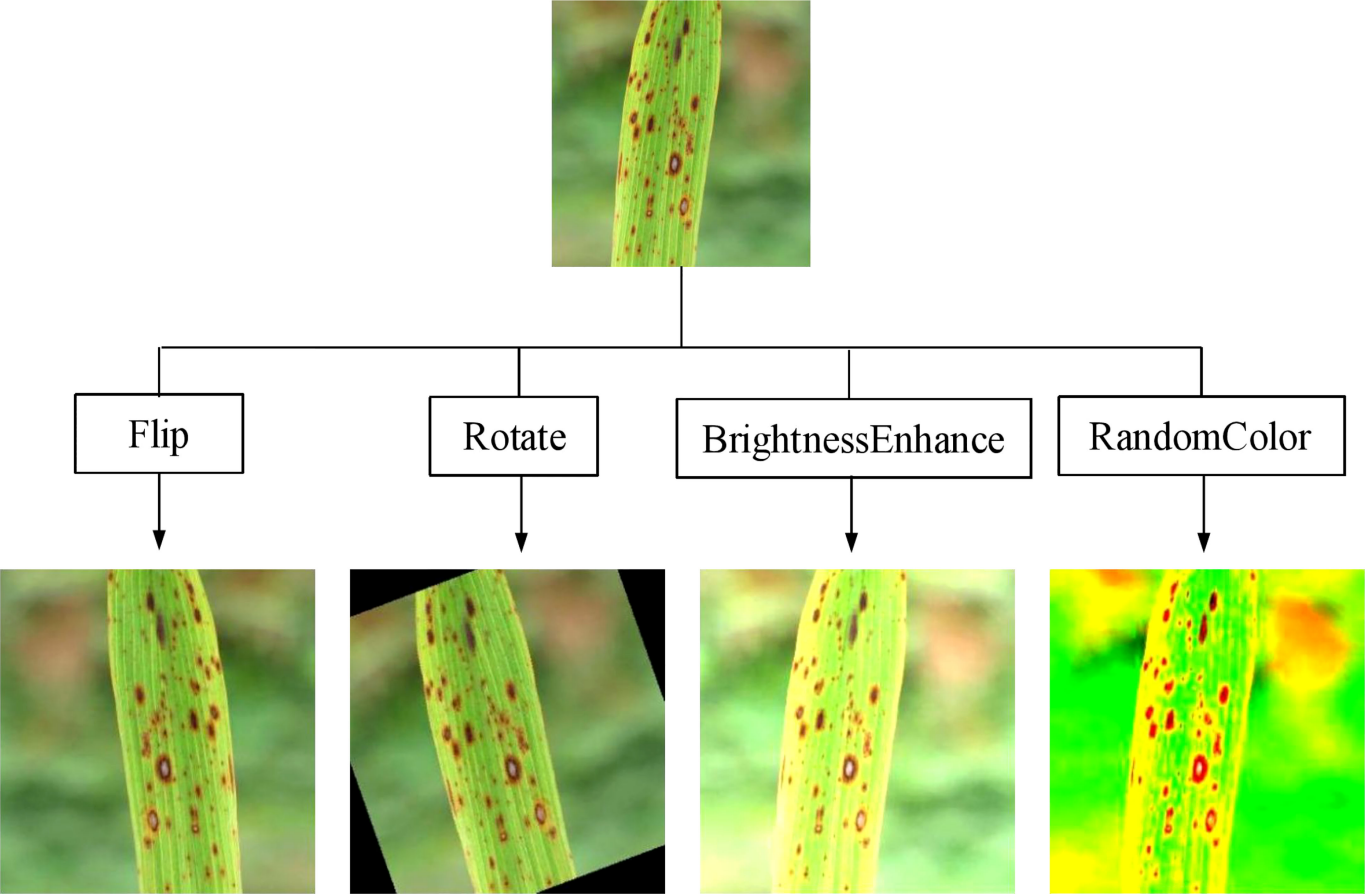
Some data enhancement renderings.

The scales of the images obtained from different sources were inconsistent, so this experiment first unified the images to a size of 224×224. In addition, deep learning generally requires a large amount of data for model training; otherwise, overfitting and poor accuracy may occur. However, under the existing conditions, a large number of rice leaf disease images could not be obtained, so the collected small dataset needed to be expanded. The original dataset was expanded using expansion methods such as flipping, rotation, cropping, color transformation, and blurring. **Figure 2** shows the effect of partially utilizing these data enhancement methods, and **Table 1** shows the numbers of images possessed before and after expansion.

**Table 1:** Dataset of rice leaf diseases.

Species	Before expansion	After expansion
Aphelenchoides Bessyi	82	1200
Bacterial Leaf Blight	200	1200
Bacterial Leaf Streak	120	1200
Brown Spot	200	1200
Leaf Smut	200	1200
Red Blight	95	1200
Rice Blast	125	1200
Rice SheathBlight	100	1200

Finally, 80% of the expanded dataset was randomly selected as the training set, and the remaining 20% was used as the test set. The training set was used again to train the model *via* the 5-fold cross-validation method, and the rice leaf disease image samples were labeled with a two-dimensional one-hot coded label array, where 0, 1, 2, 3, 4, 5, 6 and 7 represented dry tip nematodes, white leaf blight, bacterial streaks, hoary leaf spots, rice leaf black spikes, red blight, rice blasts, and stripe blight, respectively.

## 3 Rice leaf disease identification model construction

### 3.1 Improvement of the AlexNet model

The original AlexNet (Krizhevsky et al., 2012), a convolutional neural network model, used the ImageNet (Deng et al., 2009) dataset, and the rice leaf disease dataset used in this study differs greatly from ImageNet. The direct application of the original model to rice leaf disease recognition would lead to a lower recognition rate. To improve the effective feature extraction and generalization ability of the model, make the model more adaptable to the dataset used in this experiment, and further improve its recognition effect after repeated debugging and optimization, the original AlexNet model was improved, and the new model is named AlexNet_G. The specific improvements are as follows.

1) Activation function

In this study, the rectified linear unit (ReLU) activation function (Liang and Xu, 2021) was replaced by the Leaky ReLU activation function (Andrew et al., 2013), which prevented the output of the ReLU function from being 0 when the neuron input was negative; this might have caused that part of the neurons to not be activated and the corresponding parameters to not be updated, i.e., a “dead” neuron.

2) Network structure improvement

**Figure 3:**
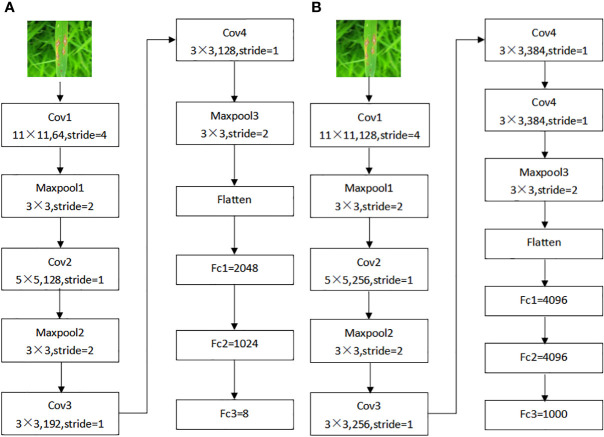
AlexNet improved structure before and after. **(A)** Original AlexNet structure, **(B)** AlexNet_G structure.

The original AlexNet model had five convolutional layers. To make the model better fit the dataset of this study, the original model was adjusted to reduce some of the convolutional layers, the number of convolutional kernels, and the number of output nodes without affecting the recognition accuracy, thus reducing the computational complexity of the model, speeding up the training process, and reducing the memory occupied by the model, as shown in **Figure 3**.

3) Optimizer improvements

In this study, the SGD (Ruder, 2016) optimizer was changed to a self-applicable learning rate-based Adam (Ruder, 2016) optimizer, which automatically adjusted the parameter learning rate, dramatically increasing the training speed and robustness of the model.

### 3.2 Improvement of the GoogLeNet model

**Figure 4:**
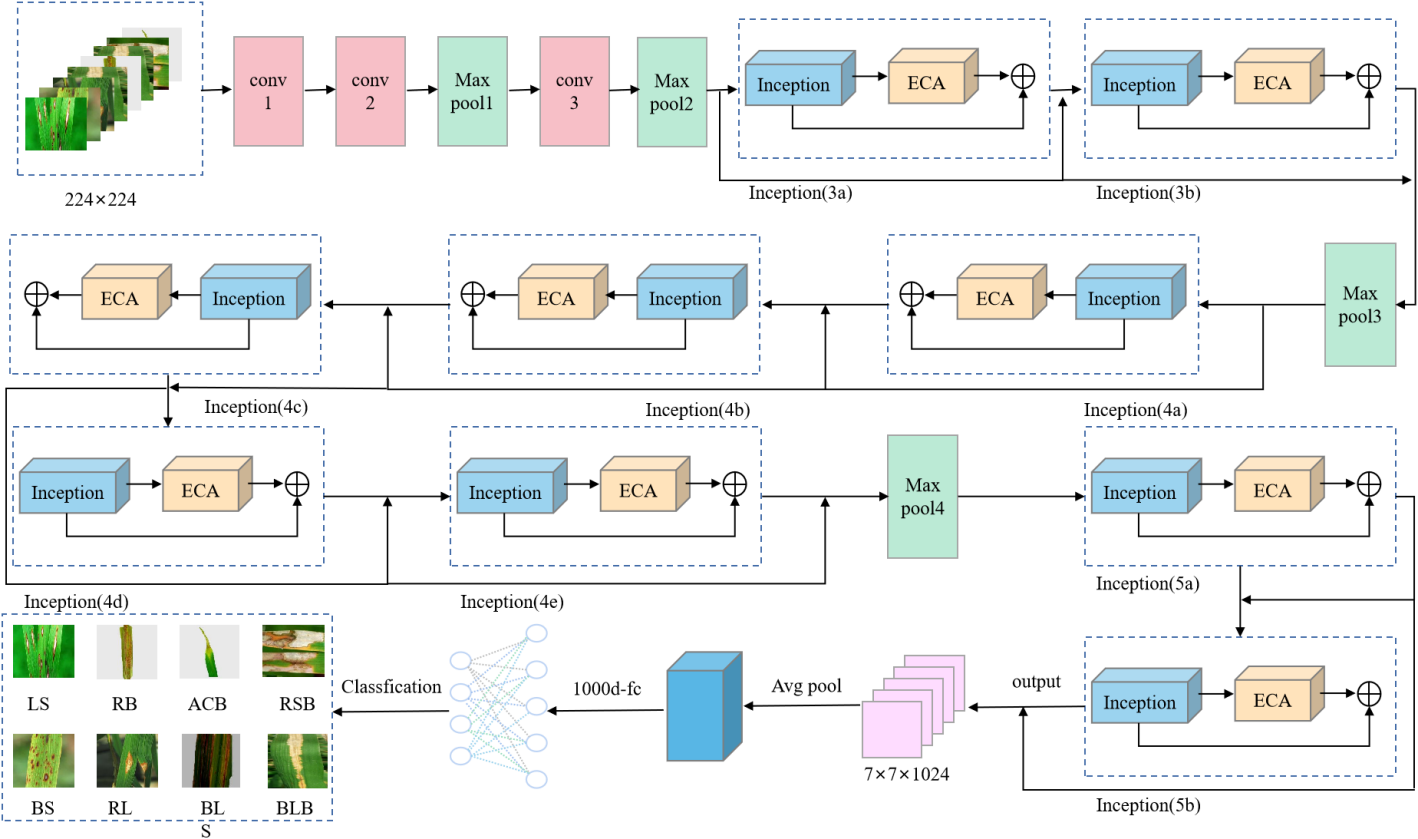
RE-GoogLeNet structure.

In 2014, the GoogLeNet model was proposed by Google (Szegedy et al., 2014), which provided a structural innovation while increasing the depth of the network by introducing a structure called Inception to replace the previous classic convolution-plus-activation component. The top-5 error rate of GoogLeNet on the ImageNet classification competition was reduced to 6.7%. In this study, the ECA mechanism and a residual network were added to GoogLeNet, the new obtained model is named RE-GoogLeNet (Yang et al., 2023); its structure is shown in **Figure 4**, with the following improvements.

1) Activation function

In this study, the Leaky ReLU activation function replaced the ReLU activation function so that the extraction rate of rice leaf disease features could be improved and the sparsity of the ReLU activation function could be reduced.

2) Convolution kernel

The 7×7 convolutional kernels in the original GoogLeNet were replaced with three 3×3 convolutional kernels to introduce more nonlinearity.

3) Embedding the ECA attention mechanism in the Inception module

**Figure 5:**
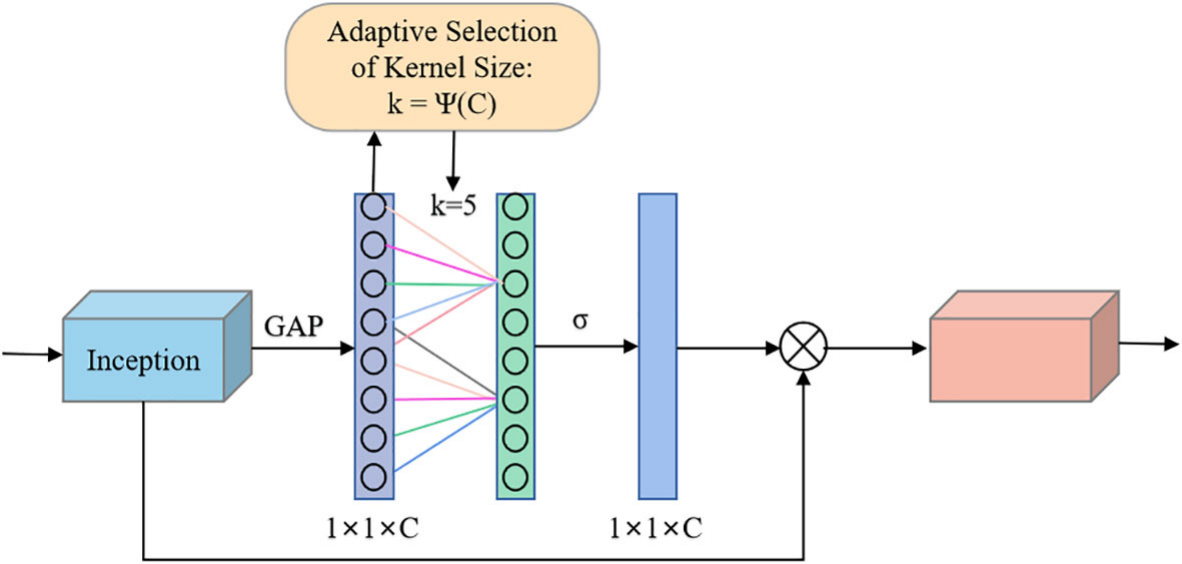
E-Inception structure.

The incorporation of attention mechanisms into convolutional neural networks to improve the performance of plant leaf disease recognition has become a major hotspot and has been shown to be beneficial for improving model performance. In this experiment, the ECA mechanism (Wang et al., 2020) was selected and added to the Inception module in GoogLeNet, which is called the E-Inception module, as shown in **Figure 5**. The specific structure adds a fully connected layer after the Inception module, connects a one-dimensional convolution with adaptive channels, and finally uses the sigmoid activation function to output the feature map.

4) Addition of residual connections

**Figure 6:**
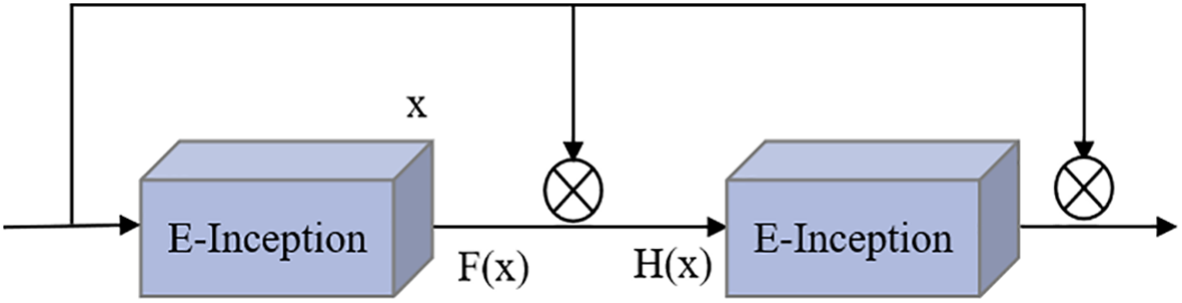
Res-ECA-Inception module.

Adding the attention mechanism after the Inception block could enable the extraction of deeper feature information, but after adding the ECA mechanism, the obtained E-Inception had more network layers than the previous module. This increase in the number of network layers would increase the information loss and gradient loss. In 2015, Kaiming He et al. proposed the ResNet network (He et al., 2016), and the increase in the number of network layers increased the information loss and elevation loss. Therefore, this study added a residual network between each pair of E-Inception layers and added a bypass between layers with the same performance to linearly superimpose the feature information of the previous E-Inception layer and the feature output of this layer to mitigate the degradation of the weight matrix when training the deep neural network. This approach is named the Res-ECA-Inception module, and the specific connection method is shown in **Figure 6**.

### 3.3 Ensemble learning

**Figure 7:**
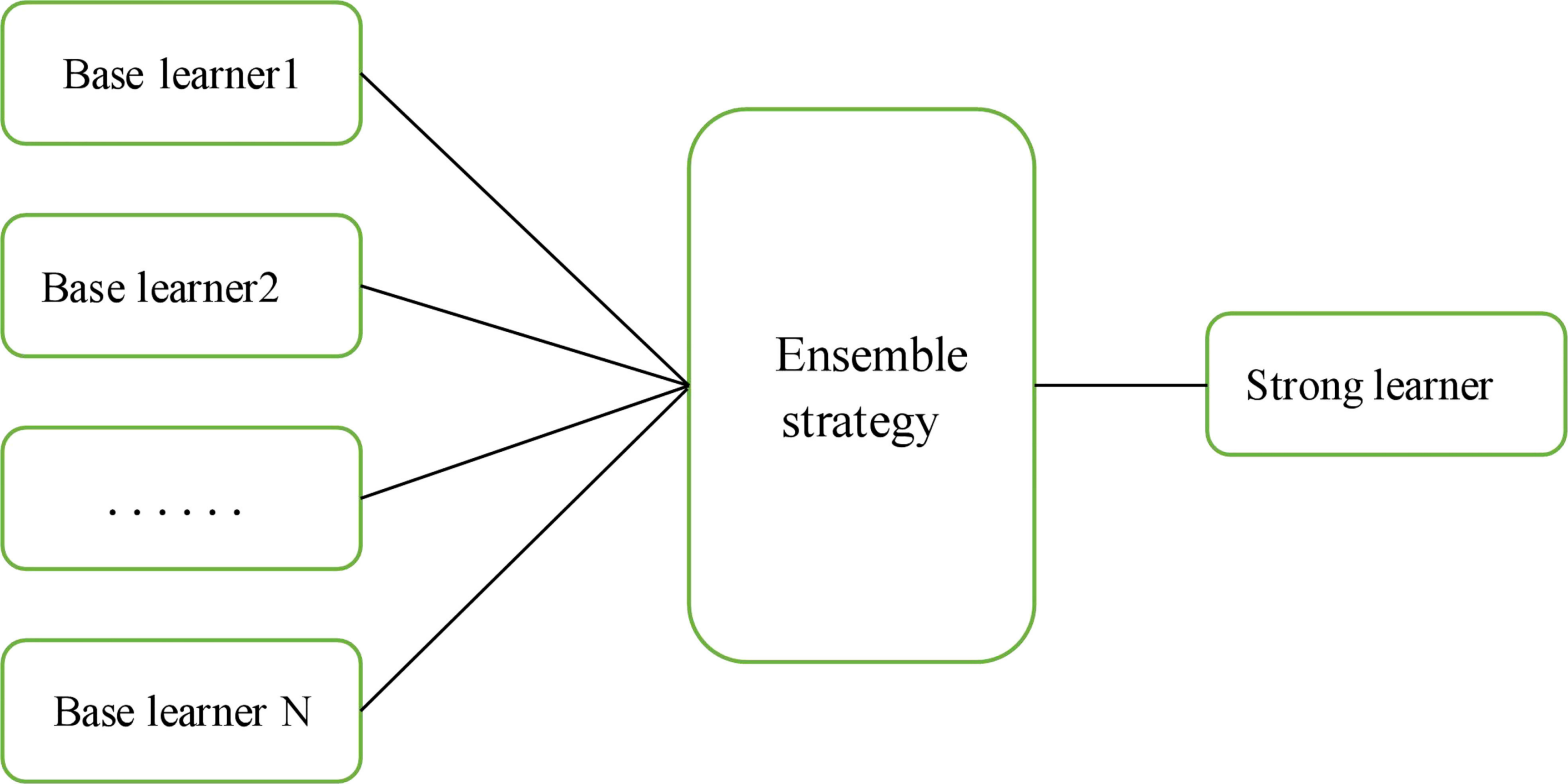
Ensemble Learning Overview.

Ensemble learning involves constructing and combining several learners to complete a learning task; this approach is sometimes referred to as a multiclassifier system, committee-based learning, etc. **Figure 7** shows a generalization of the idea of integrated learning. The learning task is accomplished by training several individual learners with certain combination strategies to eventually form a strong learner.

**Figure 8:**
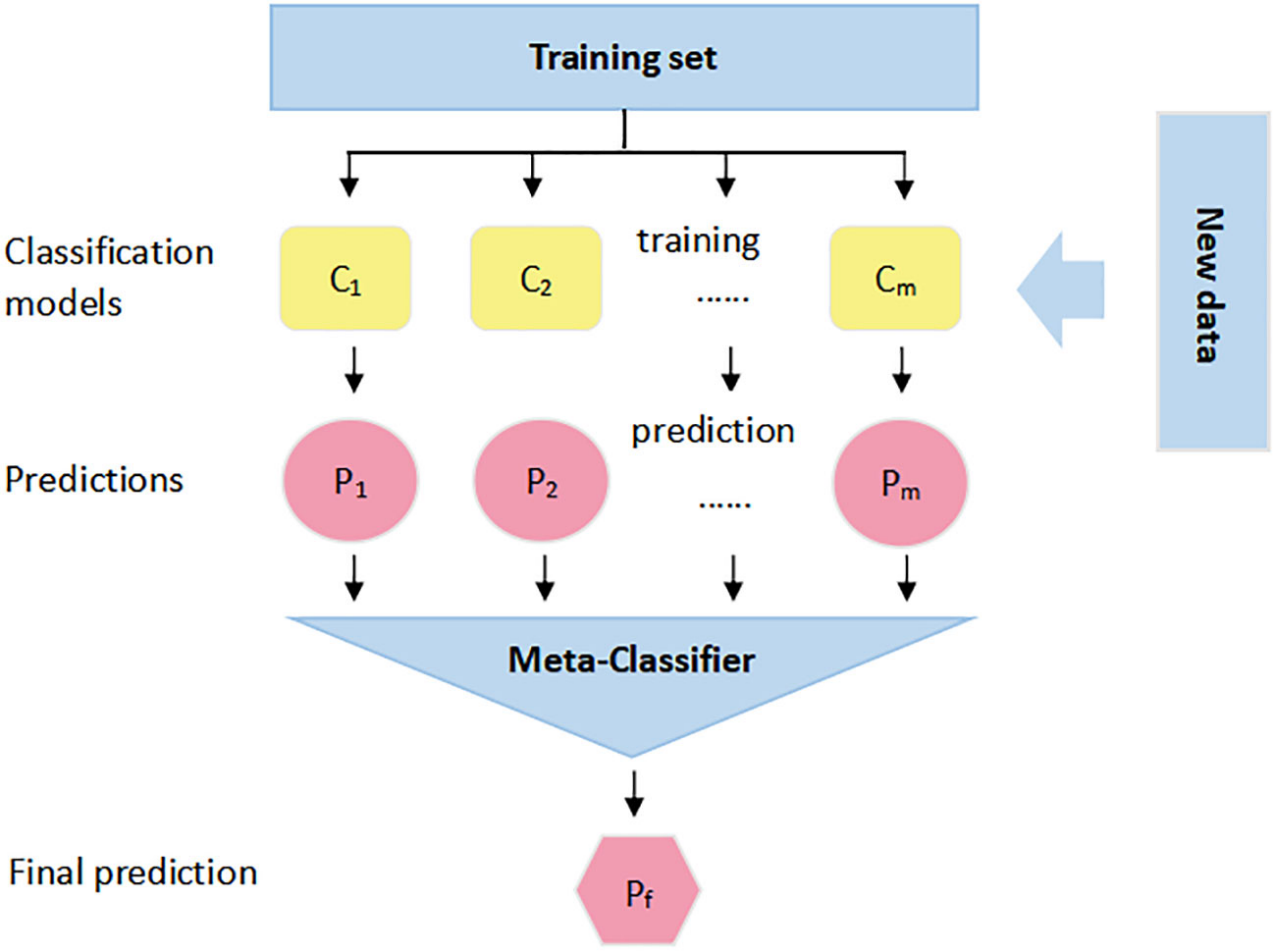
Stacking model fusion process.

The main ensemble learning methods include bagging, boosting, and stacking, and the stacking method was used in this study. The stacking method, first proposed by Wolpert (Wolpert, 1990), is a serially structured hierarchical stacking ensemble framework that is popular in major data mining competitions. It consists of two levels of classifiers, called base learners and secondary learners, using a learning strategy for model fusion; i.e., the secondary learners are retrained and predicted using the outputs of the base learners as features to obtain complete predictions for correcting several base learner errors, thus actively improving the integrated model performance and reducing the risk of overfitting. Ensemble learning is widely used in education, medicine, social sciences, etc., but it has been less used in agriculture. The stacking model fusion process is shown in **Figure 8**. C_1_,…, C_m_ are the base classifiers (base models), and each base classifier’s training set is the complete original training set. For each base classifier, N epochs are used for training, and after the training process, all outputs (P_1_,…, P_m_) of (C_1_,…, C_m_) for the original training set during the N epochs are combined as a new training set for the second training stage of the model-meta classifier.

**Figure 9:**
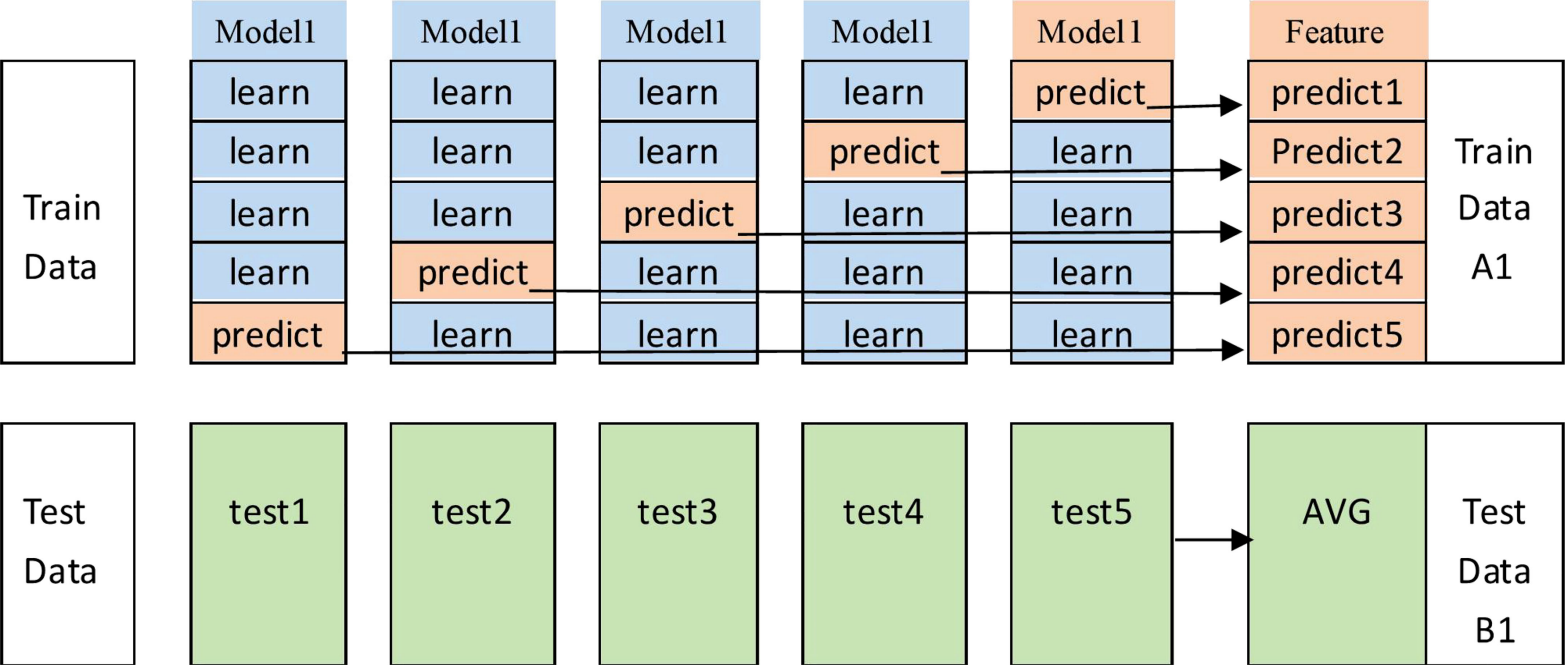
5-fold cross-validation process in stacking.

In the actual use of the stacking method, to avoid the risk of overfitting, it is often accompanied by a cross-validation operation, and this study used a 5-fold cross-validation. The specific process is shown in **Figure 9**. The original dataset is first divided into a training set (the training data in **Figure 9**) and a test set (the test data in **Figure 9**) using 5-fold cross-validation; i.e., the original training set is divided into five folds, among which four folds are recorded as the base learner training set (“learn”), and the remaining one fold is recorded as the validation set (“predict”). The cross-validation process consists of two parts, i.e., a stable model is obtained by training on the dataset, and then the model is used for prediction.

1) The base learner is used to train on the learning folds and perform prediction on “predict” to output the feature column predict1; the test set is used to train this stereotyped parameter learner, and the prediction result test1 is output.
2) The above steps are repeated 5 times to generate predict1, predict2,…,predict5 from the training data, and these results are merged vertically to obtain A1. The test data generate test1, test2,…, test5, and these are averaged them to obtain B1.
3) The same operation is performed for the other base learners, i.e., repeating steps 1 and 2. Suppose we have four base learners; then, A1, A2, A3, and A4 and B1, B2, B3, and B4 are generated.
4) A1, A2, A3, and A4 are input as training sets into the stacking sublearner for training, and then the trained fixed-parameter models are tested against B1, B2, B3, and B4 to obtain the final results.

This implements the stacking method in a 5-fold cross-validation manner.

### 3.4 The experimental model

In this study, the AlexNet_G, RE-GoogLeNet, ResNet50, and MobileNetV3 (Howard et al., 2019) models were used as the base learners of the stacking ensemble learning framework so that the fusion models could be optimized and improved from the base models with large differences.

## 4 Experimental results and discussion

This experiment used a computer with an Intel(R) Xeon(R) Silver 4112 CPU at 2.60 GHz, 64.0 GB of RAM, an NVIDIA Quadro RTX 5000 graphics card; CUDA version 10.1; Cudnn version 7.6.5; and the Windows 10 64-bit operating system. The utilized software mainly included the OpenCV image processing software and the TensorFlow 2.3 Python 3.7 deep learning framework, using the Python language for program compilation.

### 4.1 Evaluation indicators

In this study, the precision, recall, accuracy, and F1 metrics were used to measure the performance of the network model in terms of rice leaf disease identification. The precision, recall, accuracy and F1 evaluation metrics were calculated as follows.


(3) $$\text{Precision}=\frac{\text{TP}}{\text{TP}+\text{FP}}$$

(4) $$\text{Recall}=\frac{\text{TP}}{\text{TP}+\text{FN}}\;$$

(5) $$\text{ Accuray}=\frac{\text{TP}+\text{TN}}{\text{TP}+\text{FN}+\text{FP}+\text{TN}}\;$$

(6) $$\text{F}1=\frac{2\text{TP}}{2\text{TP}+\text{FP}+\text{FN}}\;$$

where TP indicates that a positive sample was predicted as a positive sample, that is, a correct prediction; FP indicates that a negative sample was predicted as a positive sample, that is, an incorrect prediction; FN indicates that a positive sample was predicted as a negative sample, that is, an incorrect prediction; and TN indicates that a negative sample was predicted as a negative sample, that is, a correct prediction. The accuracy rate, also called the check rate, aims to determine how many of the samples predicted to be positive are actually positive and is used to evaluate the correctness of the detector based on successful detections. The recall rate, also called the check all rate, aims to find how many of the actually positive samples are predicted to be positive and is used to evaluate the detection coverage of the detector for all targets to be detected. The accuracy rate aims to know the probability of correct prediction among the total samples. F1 is designed to reflect both the accuracy rate and recall rate as an evaluation metric.

### 4.2 Experiments on open datasets

**Figure 10:**
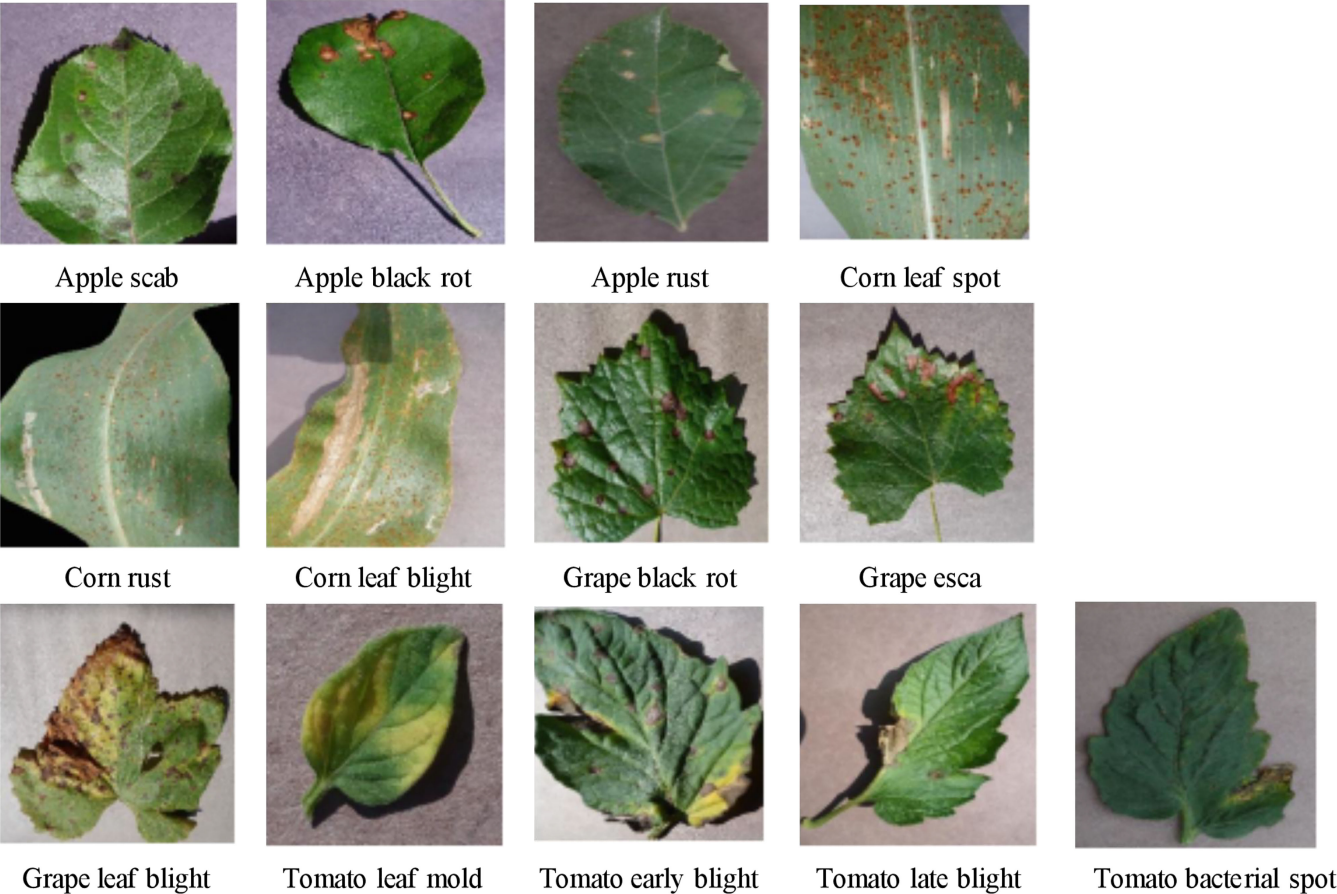
Plant disease pictures.

Considering the small size of the original rice leaf dataset, the four varieties of disease images were selected from the PlantVillage public dataset as the experimental subjects to verify the generalization ability of the model proposed in this study. These included apple black scab, apple black rot, apple rust, corn leaf spot, corn rust, corn leaf blight, grape black rot, grape esca, grape leaf blight, tomato leaf mold, tomato early blight, tomato late blight, and tomato bacterial spot. Some of these are shown in **Figure 10**, with a total of 15,866 images, and the numbers of images in various categories are shown in **Table 2**. As in the experiments conducted on the rice leaf dataset, we used 20% of the original dataset for evaluating the model, and the other 80% was still used for training and testing the model using fivefold cross-validation.

**Table 2:** The number of different plant diseases.

Species	No.	Plant disease	Number
Apple	1	Apple scab	1000
	2	Apple black	1000
	3	Apple rust	1000
Corn	4	Corn leaf spot	1000
	5	Corn rust	1192
	6	Corn leaf blight	1000
Grape	7	Grape black rot	1180
	8	Grape esca	1383
	9	Grape leaf blight	1076
Tomato	10	Tomato bacterial spot	2127
	11	Tomato early blight	1000
	12	Tomato late blight	1908
	13	Tomato leaf mold	1000

### 4.3 Comparisons before and after improving the model

In this study, the improved AlexNet and GoogLeNet were used for the model. To explore the performance of the improved models, the single models were separately trained and tested on the rice leaf disease dataset and plant disease dataset. Their performance was measured using different evaluation metrics, such as the accuracy, precision, recall and F1 measures, and the data here were the average values in the fivefold cross-validation process. The results are shown in **Tables 3**, **4**. From the data in the tables, we can see that the improved AlexNet had a better recognition ability than the original model on both the rice disease dataset and the plant disease dataset, with average accuracy improvements of 0.36% and 1.66%, respectively, and the improved AlexNet had fewer parameters. The improved GoogLeNet also achieved better performance on the rice disease dataset and plant disease dataset, with average accuracy improvements of 0.42% and 2.34%, respectively, over the original model. The other metrics of both models were also improved.

**Table 3:** Improved model for rice disease recognition results.

Model	Accuracy%	Precision%	Recall%	F1-score%
AlexNet	97.76	97.77	97.76	97.76
AlexNet_G	98.12	98.13	98.11	98.12
GoogLeNet	97.81	97.84	97.84	97.83
RE-GoogLeNet	98.23	98.22	98.23	98.22

**Table 4:** Improved model for plant disease recognition results.

Model	Accuracy%	Precision%	Recall%	F1-score%
AlexNet	92.83	92.61	92.36	92.42
AlexNet_G	94.49	94.38	94.10	94.20
GoogLeNet	90.02	89.96	89.61	89.63
RE-GoogLeNet	92.36	92.11	92.24	92.08

### 4.4 Comparison between different sublearners

To determine the effects of different sublearners on the performance of the stacking-based integrated model, seven more common classification algorithms in machine learning were selected in this study, namely, an SVM, k-nearest neighbors (KNN) (Tang, 2013), a random forest (RF) (Abeywickrama et al., 2016), GNB, a decision tree (DT), a light gradient boosting machine (LGBM), and LGR. With the same hyperparameters for each model, the rice dataset and the plant dataset were used for training, and the obtained accuracy rates are shown in **Table 5**. Finally, it was found that the accuracy rates achieved on both datasets were maximized when using the SVM, so in this study, the SVM was used as the sublearner in the stacking ensemble learning model.

**Table 5:** Comparison of results from different secondary learners.

Data set	SVM	KNN	RF	GNB	DT	LGBM	LGR
Rice disease dataset	99.69%	99.22%	99.58%	98.23%	98.49%	99.38%	99.53%
Plant disease dataset	99.15%	98.52%	98.99%	95.12%	97.95%	98.99%	99.09%

### 4.5 Single-model analysis

**Figure 11:**
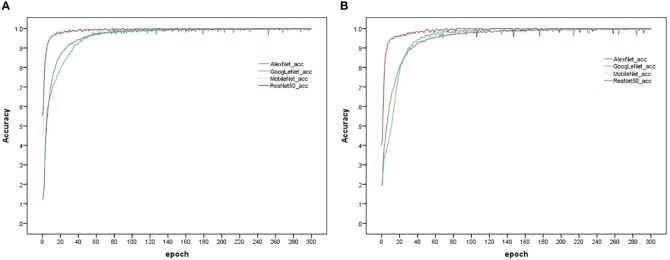
Accuracy variation of the four models on the training set. **(A)** Rice disease dataset, **(B)** Plant disease dataset.

The model training and testing processes in this study were performed under the TensorFlow framework, and the rice dataset and plant dataset were used to train AlexNet_G, RE-GoogLeNet, ResNet50 and MobileNetV3 separately. All hyperparameters were kept consistent to ensure that the models were trained under the same environment, and the training results are shown in **Figure 11**. From (a) and (b) of **Figure 10**, it is observed that the model accuracy improvement occurred gradually as the number of training sessions increased and finally stabilized, which indicates that the model was better trained. The final accuracies of AlexNet_G, RE-GoogLeNet, ResNet50, and MobileNetV3 on the training set of the rice dataset were stable at 98.12%, 98.23%, 98.29%, and 92.89%, respectively, and on the training set of the plant dataset, they were stable at 94.49%, 92.36%, 97.23%, and 93.31%, respectively.

Finally, the single models were validated with images from the test set, and their classification accuracies were calculated by calculating the numbers of correctly predicted rice leaf diseases and plant diseases. In addition, the precision, recall and F1 values were also calculated, and each value is shown in **Tables 6**, **7**.

**Table 6:** Comparison of results from different single models (Rice disease dataset).

Models	Recognized quantity	Correct quantity	Mistak quantity	Accuracy%	Precision%	Recall%	F1-score%
AlexNet_G	1920	1884	36	98.12	98.16	98.11	98.13
RE-GoogLeNet	1920	1886	34	98.23	98.21	98.23	98.21
ResNet50	1920	1887	33	98.29	98.30	98.21	98.22
MobileNetV3	1920	1784	136	92.89	92.92	92.86	92.86

**Table 7:** Comparison of results from different single models (Plant disease dataset).

Models	Recognized quantity	Correct quantity	Mistake quantity	Accuracy%	Precision%	Recall%	F1-score%
AlexNet_G	3174	2999	175	94.49	94.53	94.54	94.52
RE-GoogLeNet	3174	2932	242	92.36	92.09	92.37	92.16
ResNet50	3174	3086	88	97.23	97.27	97.04	97.14
MobileNetV3	3174	2962	211	93.31	93.39	93.27	93.33

### 4.6 Combination models with different base learners

In this experiment, the AlexNet_G, RE-GoogLeNet, ResNet50 and MobileNetV3 models were arbitrarily combined and fused into new stacking-based integrated models to explore the performance changes yielded by integrated models with different combinations, and the sublearner was an SVM. Consistent with the previous single-model validation method, the classification accuracy was calculated according to the numbers of correct rice leaf disease and plant disease classifications. Other model evaluation metrics were also employed. The test accuracies of the stacking models with different base learner combinations for the same rice leaf disease and plant disease datasets are shown in **Tables 8**, **9**, where A denotes AlexNet_G, G denotes RE-GoogLeNet, R denotes ResNet50, and M denotes MobileNetV3.

**Table 8:** Comparison of results of different base learner combinations (Rice disease dataset).

Base learner combination	Recognized quantity	Correct quantity	Mistake quantity	Accuracy%	Precision%	Recall%	F1-score%
AR	1920	1910	10	99.48	99.45	99.48	99.47
AG	1920	1901	19	99.11	99.12	99.11	99.11
AM	1920	1900	20	98.80	98.90	98.89	98.91
GR	1920	1909	11	99.43	98.17	98.16	98.17
GM	1920	1902	18	99.06	96.50	96.47	96.50
RM	1920	1899	21	98.90	98.12	98.11	98.12
AGR	1920	1913	7	99.62	97.22	97.23	97.22
AGM	1920	1907	13	99.32	97.15	97.14	97.15
GRM	1920	1910	10	99.47	98.16	98.19	98.16
AGRM	1920	1914	6	99.69	98.96	98.94	98.96

**Table 9:** Comparison of results of different base learner combinations (Plant disease dataset).

Base learner combination	Recognized quantity	Correct quantity	Mistake quantity	Accuracy%	Precision%	Recall%	F1-score%
AR	3174	3126	48	98.33	98.31	98.31	98.33
AG	3174	3072	102	96.68	96.60	96.62	96.68
AM	3174	3077	97	96.92	96.91	96.93	96.92
GR	3174	3121	53	98.19	98.17	98.16	98.19
GM	3174	3064	110	96.42	96.50	96.47	96.42
RM	3174	3120	54	98.15	98.12	98.11	98.15
AGR	3174	3086	88	97.23	97.22	97.23	97.23
AGM	3174	3086	88	97.17	97.15	97.14	97.17
GRM	3174	3123	51	98.30	98.16	98.19	98.30
AGRM	3174	3145	29	99.15	98.94	98.96	98.94

From the table, we can see that the results obtained using the stacking ensemble models were at high levels; the accuracies achieved on the rice leaf disease dataset were all above 99% except for those of two combinations, AM and RM, which did not reach 99% accuracy. All other combinations exceeded 99% accuracy on the plant disease dataset. The stacking-based integrated model also achieved good results, but not on the rice leaf disease dataset. The reason for the lower accuracy yielded on this dataset may be that there are few original pictures of rice, and the dataset obtained *via* data expansion leads to high accuracy. However, the stacking-based integrated model was more effective than a single model on both datasets. On the rice leaf disease dataset, the accuracy of AlexNet_G plus MobileNetV3 was 98.80%, making this the least effective among all combined stacking models but still 0.51% more accurate than the best-performing single model (ResNet50). This is because a single classifier can fall into local optima and induce overfitting during training for various reasons, resulting in a poor model generalization ability, while the stacking-based integrated model integrates the performance of multiple individual classifiers, effectively reducing or avoiding the aforementioned risk, thus enhancing its generalization ability and improving the accuracy of rice leaf classification recognition. **Tables 10**, **11** show the accuracy, recall and F1 values obtained for each disease in the rice dataset and plant dataset, respectively.

**Table 10:** The recognition results of different rice disease dataset.

Type names	Precision%	Recall%	F1-score%
AphelenchoidesBessyi	99.60	100	99.80
BacterialLeafBlight	100	100	100
BacterialLeafStreak	100	99.57	99.78
BrownSpot	100	100	100
LeafSmut	99.56	100	99.78
RedBlight	98.76	100	99.37
RiceBlast	100	98.76	99.38
RiceSheathBlight	99.15	98.72	98.93

**Table 11:** The recognition results of different plant disease dataset.

Type names	Precision%	Recall%	F1-score%
Apple scab	100	100	100
Apple black	100	100	100
Apple rust	100	100	100
Corn leaf spot	92.48	96.31	94.36
Corn rust	100	100	100
Corn leaf blight	95.68	91.24	93.40
Grape black rot	100	100	100
Grape esca	100	100	100
Grape leaf blight	100	100	100
Tomato bacterial spot	100	99.76	99.88
Tomato early blight	98.07	100	99.02
Tomato late blight	100	99.20	99.60
Tomato leaf mold	100	100	100

### 4.7 Comparison with other models

To validate the classification performance of the model proposed in this study, the same approach as above was taken to further train and test the model on the rice disease dataset and plant disease dataset. The more influential convolutional neural networks, which include DenseNet121 (Huang et al., 2017), InceptionResNetV2 (Szegedy et al., 2017), InceptionV3 (Szegedy et al., 2016), ResNet34, and ResNet101, were selected in this paper, and the performances achieved by the models on the rice dataset and the plant dataset are shown in **Tables 12**, **13**, respectively.

**Table 12:** The recognition results of different models(Rice disease dataset).

No.	Models	Traing Accuracy%	Test Accuracy%	F1-score%
1	DenseNet121	98.79	98.91	98.80
2	InceptionReaNetV2	98.96	99.08	98.96
3	InceptionV3	98.62	98.78	98.63
4	ResNet34	97.74	98.10	97.74
5	ResNet101	98.07	98.52	98.08
6	This study	99.42	99.69	99.63

**Table 13:** The recognition results of different models(Plant disease dataset).

No.	Models	Traing Accuracy%	Test Accuracy%	F1-score%
1	DenseNet121	98.77	98.49	98.38
2	InceptionResNetV2	97.97	97.89	97.77
3	InceptionV3	98.13	97.99	97.84
4	ResNet34	96.45	96.29	96.01
5	ResNet101	97.91	97.94	97.84
6	This study	99.08	99.15	98.95

**Figure 12:**
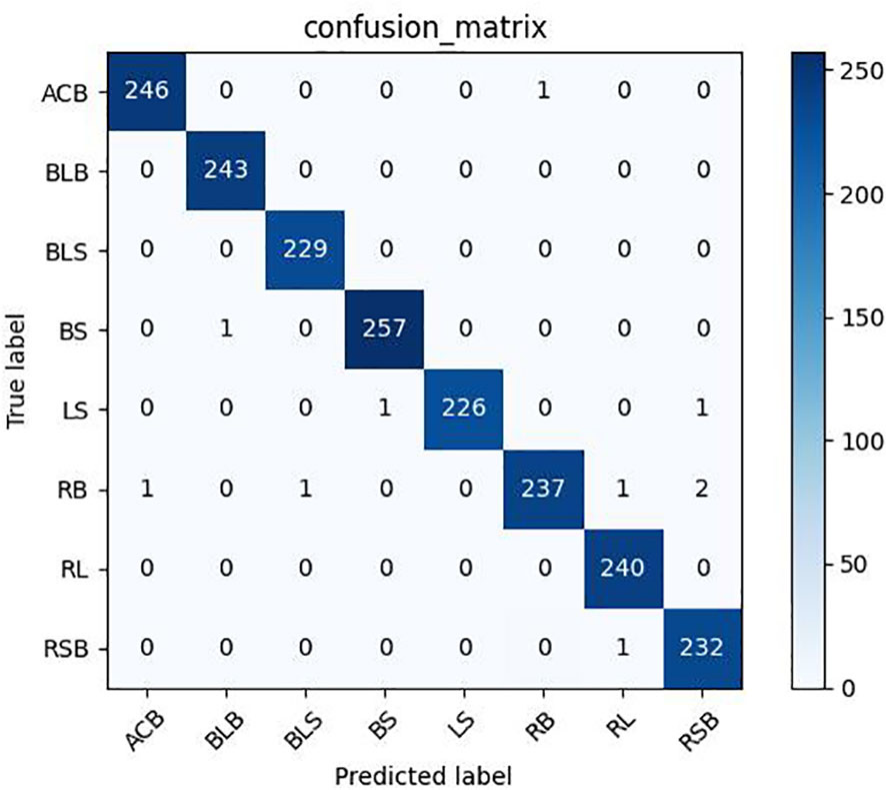
Confusion matrix for rice dataset.

**Figure 13:**
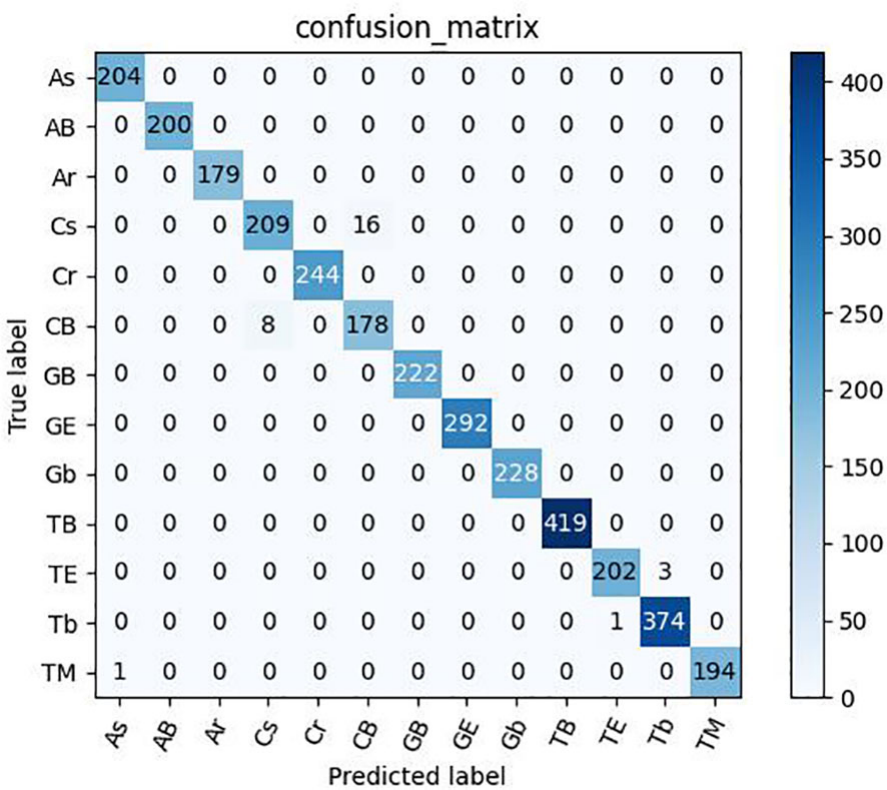
Confusion matrix for Plant dataset.

As shown in **Tables 12**, **13**, the model proposed in this study successfully achieved improved performance over that of other advanced methods, with testing accuracies of 99.69% and 99.15% on the rice dataset and the plant dataset, respectively. Among them, DenseNet121 and InceptionResNetV2 are both deep convolutional neural networks, but the model proposed in this paper still yielded better results than these two techniques. In addition, **Figures 12**, **13** show the confusion matrices obtained from model training on both datasets, and it is shown that our proposed model successfully identified the majority of the crop disease types in each sample image. Among them, As is apple black scab, AB is apple black rot, Ar is apple rust, Cs is corn leaf spot, Cr is corn rust, CB is a corn leaf blight, GB is grape black rot, GE is grape esca, Gb is grape leaf blight, TB is tomato bacterial spot, TE is tomato early blight, Tb is tomato late blight, and TM is tomato leaf mold. In summary, the superiority of the proposed method in terms of performance has been demonstrated, and the method is also applicable to disease identification for other crops.

## 5 Conclusion

Timely and accurate crop disease identification is essential for improving the quantities and yields of crops. Deep learning techniques can be effective for image classification as they address most of the technical challenges associated with crop disease identification (Barbedo, 2019). By exploring the functions of currently popular convolutional networks, this study proposes a new network architecture. Considering the high similarity between rice leaf diseases, we use Stacking ensemble learning method to integrate convolutional neural networks together and add attention mechanism to make the model more focused on the disease part. This enables the model to better extract global features of rice leaves, and finally uses SVM for classification, with an accuracy rate of 99.69%. The proposed method was shown to be remarkably effective in identifying various crop diseases.

By applying this model to other plant datasets and achieving good results, it indicates that our model has strong generalization ability. Train the model using different sub learners to find the most suitable classifier for this model, and further prove that the combination of the proposed model achieves the best performance by comparing models with different combinations of base learners. In addition, compared to other most advanced convolutional networks, it achieved competitive performance, although the training process is slightly complicated. In the next step, further simplification of the training process and improving the efficiency of the model will be considered. In addition, we plan to deploy the model on portable devices to automatically track and identify a wide range of knowledge related to crop diseases. And applied in other fields, such as classification and recognition in animals, automobiles, and other fields.

## Data availability statement

The raw data supporting the conclusions of this article will be made available by the authors, without undue reservation.

## Author contributions

HL was responsible for the experiment design. YZ prepared materials. SX and HZ performed the program development. XY, LY, and SZ analyzed the data, and XY were responsible for writing the manuscript. LY and XY contributed to reviewing the manuscript. All authors contributed to the article and approved the submitted version. 
